# Aqua­azido­{2,2′-[*o*-phenylenebis(nitrilo­methyl­idyne)]diphenolato}manganese(III) hemihydrate

**DOI:** 10.1107/S1600536809020200

**Published:** 2009-06-06

**Authors:** Xiutang Zhang, Peihai Wei, Bin Li, Chunyong Wu, Bo Hu

**Affiliations:** a Advanced Material Institute of Research, Department of Chemistry and Chemical Engineering, Shandong Institute of Education, Jinan 250013, People’s Republic of China; b College of Chemistry and Chemical Engineering, Liaocheng University, Liaocheng 252059, People’s Republic of China; c Yuncheng Normal School, Yuncheng 274700, Shandong Province, People’s Republic of China

## Abstract

In the title compound, [Mn(C_20_H_14_N_2_O_2_)(N_3_)(H_2_O)]·0.5H_2_O, the Mn^III^ ion is chelated by the *N*,*N*′,*O*,*O*′-tetra­dentate Schiff base ligand and further coordinated by one azide ion and one water mol­ecule in *trans* positions, resulting in a distorted *fac*-MnN_3_O_3_ octa­hedral arrangement. The O atom of the uncoordinated water mol­ecule lies on a crystallographic twofold axis. In the crystal, O—H⋯O and O—H⋯N hydrogen bonds help to establish the packing.

## Related literature

For background to salicylaldehyde complexes, see: Alam *et al.* (2003 ▶); Zelewsky & von Knof (1999 ▶).
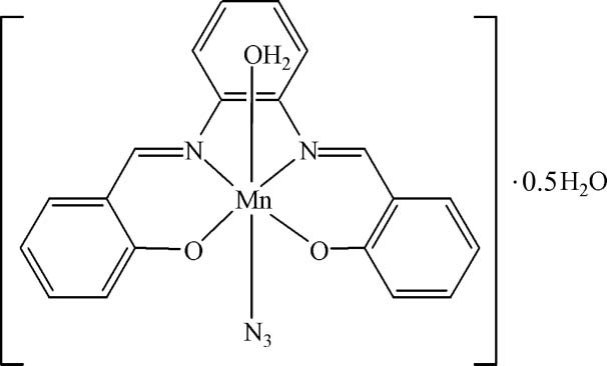


## Experimental

### 

#### Crystal data

[Mn(C_20_H_14_N_2_O_2_)(N_3_)(H_2_O)]·0.5H_2_O
*M* *_r_* = 438.33
Monoclinic, 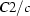

*a* = 25.100 (10) Å
*b* = 11.478 (5) Å
*c* = 12.599 (5) Å
β = 94.175 (3)°
*V* = 3620 (3) Å^3^
*Z* = 8
Mo *K*α radiation
μ = 0.77 mm^−1^
*T* = 293 K
0.12 × 0.10 × 0.08 mm

#### Data collection

Bruker APEXII CCD area-detector diffractometer
Absorption correction: multi-scan (*SADABS*; Bruker, 2004 ▶) *T*_min_ = 0.914, *T*_max_ = 0.941
11927 measured reflections
3162 independent reflections
2371 reflections with *I* > 2σ(*I*)
*R*_int_ = 0.082

#### Refinement

*R*[*F* ^2^ > 2σ(*F* ^2^)] = 0.037
*wR*(*F* ^2^) = 0.086
*S* = 1.00
3162 reflections
280 parameters
4 restraints
H atoms treated by a mixture of independent and constrained refinement
Δρ_max_ = 0.32 e Å^−3^
Δρ_min_ = −0.38 e Å^−3^

### 

Data collection: *APEX2* (Bruker, 2004 ▶); cell refinement: *SAINT-Plus* (Bruker, 2004 ▶); data reduction: *SAINT-Plus*; program(s) used to solve structure: *SHELXS97* (Sheldrick, 2008 ▶); program(s) used to refine structure: *SHELXL97* (Sheldrick, 2008 ▶); molecular graphics: *SHELXTL* (Sheldrick, 2008 ▶); software used to prepare material for publication: *SHELXTL*.

## Supplementary Material

Crystal structure: contains datablocks global, I. DOI: 10.1107/S1600536809020200/hb2977sup1.cif

Structure factors: contains datablocks I. DOI: 10.1107/S1600536809020200/hb2977Isup2.hkl

Additional supplementary materials:  crystallographic information; 3D view; checkCIF report

## Figures and Tables

**Table 1:** Selected bond lengths (Å)

Mn1—O1	1.8636 (18)
Mn1—O2	1.8844 (18)
Mn1—N2	1.986 (2)
Mn1—N1	1.988 (2)
Mn1—N3	2.306 (2)
Mn1—O1*W*	2.321 (2)

**Table 2:** Hydrogen-bond geometry (Å, °)

*D*—H⋯*A*	*D*—H	H⋯*A*	*D*⋯*A*	*D*—H⋯*A*
O1*W*—H2*W*⋯N3^i^	0.82 (2)	2.12 (2)	2.937 (3)	176 (3)
O1*W*—H1*W*⋯O2^ii^	0.820 (11)	2.076 (6)	2.885 (3)	169 (2)
O2*W*—H3*W*⋯N5	0.82 (3)	2.18 (3)	3.000 (3)	173 (4)

Symmetry codes: (i) 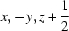; (ii) 
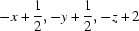
.

**Fig. 1.**
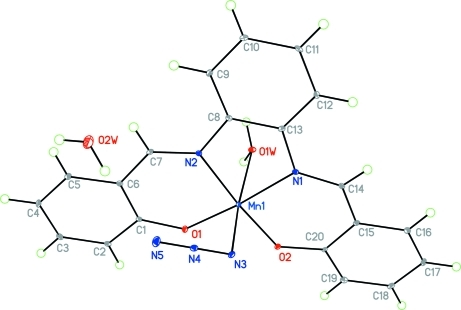
The molecular structure of (I), drawn with 30% probability displacement ellipsoids for the non-hydrogen atoms.

**Fig. 2.**
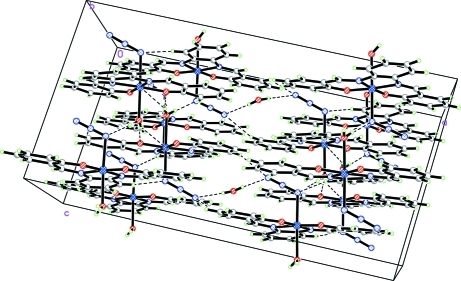
Three-dimensional network formed by hydrogen bonds (dashed lines).

## supplementary crystallographic information

### Comment

The synthesis of complexes consisting of salicylaldehyde ligand has attracted continuous research interest not only because of their appealing structural and topological novelty, but also due to their unusual optical, electronic, magnetic, and catalytic properties, as well as their potential medical application (Alam *et al.*,  2003; Zelewsky & von Knof, 1999). In the present paper, we describe the synthesis and structural characterizations of the title compound, (I),

As shown in Fig. 1, each Mn(III) atom is chelated by Schiff base ligand *via* two N and two O atoms and is additionally coordinated by one azide and a water molecule, forming a distorted octahedral geometry (Table 1) in which, the Schiff base lies in the equatorial plane, and the azide and aqua ligands lie in the axial coordination sites.

With O—H···O and O—H···N hydrogen bonds (Table 2), a three-dimensional network is formed as shown in Fig. 2.

### Experimental

A mixture of manganese(III) acetylacetonate (1 mmol) and *N*,*N*'-bis(2-hydroxy-5-bromobenzyl)1,2-diaminopropane (1 mmol), and dipotassium nickel tetracyanide (1 mmol) in 20 ml methanol was refluxed for several hours. The above cooled solution was filtered and the filtrate was kept in an ice box. One week later, brown blocks of (I) were obtained with a yield of 5%. Anal. Calc. for C_40_H_34_Mn_2_N_10_O_7_: C 54.75, H 3.88, N 15.97%; Found: C 54.71, H 3.75, N 15.82.

### Refinement

All H atoms were placed in calculated positions with C—H = 0.93Å and refined as riding with *U*_iso_(H) = 1.2*U*_eq_(carrier). H atom on aqua were located from difference density maps and were refined with distance restraints of O–H = 0.82 (1) Å.

### Crystal data

**Table:**

[Mn(C_20_H_14_N_2_O_2_)(N_3_)(H_2_O)]·0.5H_2_O	*F*(000) = 1808
*M**_r_* = 438.33	*D*_x_ = 1.612 Mg m^−^^3^
Monoclinic, *C*2/*c*	Mo *K*α radiation, λ = 0.71070 Å
Hall symbol: -C 2yc	Cell parameters from 3162 reflections
*a* = 25.10 (1) Å	θ = 3.0–25.0°
*b* = 11.478 (5) Å	µ = 0.77 mm^−^^1^
*c* = 12.599 (5) Å	*T* = 293 K
β = 94.175 (3)°	Block, pink
*V* = 3620 (3) Å^3^	0.12 × 0.10 × 0.08 mm
*Z* = 8	

### Data collection

**Table:**

Bruker APEXII CCD area-detector diffractometer	3162 independent reflections
Radiation source: fine-focus sealed tube	2371 reflections with *I* > 2σ(*I*)
graphite	*R*_int_ = 0.082
φ and ω scans	θ_max_ = 25.0°, θ_min_ = 3.0°
Absorption correction: multi-scan (*SADABS*; Bruker, 2004)	*h* = −27→29
*T*_min_ = 0.914, *T*_max_ = 0.941	*k* = −13→13
11927 measured reflections	*l* = −14→13

### Refinement

**Table:**

Refinement on *F*^2^	Primary atom site location: structure-invariant direct methods
Least-squares matrix: full	Secondary atom site location: difference Fourier map
*R*[*F*^2^ > 2σ(*F*^2^)] = 0.037	Hydrogen site location: inferred from neighbouring sites
*wR*(*F*^2^) = 0.086	H atoms treated by a mixture of independent and constrained refinement
*S* = 1.00	*w* = 1/[σ^2^(*F*_o_^2^) + (0.03*P*)^2^] where *P* = (*F*_o_^2^ + 2*F*_c_^2^)/3
3162 reflections	(Δ/σ)_max_ = 0.001
280 parameters	Δρ_max_ = 0.32 e Å^−^^3^
4 restraints	Δρ_min_ = −0.38 e Å^−^^3^

### Special details

**Table:**

Geometry. All e.s.d.'s (except the e.s.d. in the dihedral angle between two l.s. planes) are estimated using the full covariance matrix. The cell e.s.d.'s are taken into account individually in the estimation of e.s.d.'s in distances, angles and torsion angles; correlations between e.s.d.'s in cell parameters are only used when they are defined by crystal symmetry. An approximate (isotropic) treatment of cell e.s.d.'s is used for estimating e.s.d.'s involving l.s. planes.
Refinement. Refinement of *F*^2^ against ALL reflections. The weighted *R*-factor *wR* and goodness of fit *S* are based on *F*^2^, conventional *R*-factors *R* are based on *F*, with *F* set to zero for negative *F*^2^. The threshold expression of *F*^2^ > σ(*F*^2^) is used only for calculating *R*-factors(gt) *etc*. and is not relevant to the choice of reflections for refinement. *R*-factors based on *F*^2^ are statistically about twice as large as those based on *F*, and *R*- factors based on ALL data will be even larger.

### Fractional atomic coordinates and isotropic or equivalent isotropic displacement parameters (Å^2^)

**Table:**

	*x*	*y*	*z*	*U*_iso_*/*U*_eq_	
Mn1	0.204685 (15)	0.09791 (3)	0.87813 (3)	0.00903 (13)	
C1	0.11631 (10)	0.2543 (2)	0.90445 (18)	0.0102 (6)	
C2	0.09780 (10)	0.3688 (2)	0.90882 (19)	0.0124 (6)	
H2	0.1219	0.4301	0.9061	0.015*	
C3	0.04451 (10)	0.3928 (2)	0.91706 (19)	0.0156 (6)	
H3	0.0332	0.4699	0.9196	0.019*	
C4	0.00719 (10)	0.3029 (2)	0.9217 (2)	0.0180 (6)	
H4	−0.0288	0.3197	0.9260	0.022*	
C5	0.02444 (10)	0.1897 (2)	0.9198 (2)	0.0161 (6)	
H5	−0.0002	0.1297	0.9238	0.019*	
C6	0.07880 (10)	0.1623 (2)	0.91183 (18)	0.0110 (6)	
C7	0.09346 (10)	0.0426 (2)	0.91314 (18)	0.0114 (6)	
H7	0.0662	−0.0115	0.9188	0.014*	
C8	0.15275 (10)	−0.1198 (2)	0.91053 (18)	0.0092 (6)	
C9	0.11626 (10)	−0.2052 (2)	0.93648 (19)	0.0123 (6)	
H9	0.0819	−0.1844	0.9525	0.015*	
C10	0.13142 (10)	−0.3207 (2)	0.93819 (18)	0.0123 (6)	
H10	0.1071	−0.3777	0.9553	0.015*	
C11	0.18272 (10)	−0.3529 (2)	0.91454 (18)	0.0124 (6)	
H11	0.1925	−0.4311	0.9157	0.015*	
C12	0.21902 (10)	−0.2690 (2)	0.88938 (18)	0.0109 (6)	
H12	0.2533	−0.2906	0.8736	0.013*	
C13	0.20450 (10)	−0.1518 (2)	0.88751 (18)	0.0096 (5)	
C14	0.28922 (10)	−0.0719 (2)	0.84750 (19)	0.0110 (6)	
H14	0.3007	−0.1480	0.8385	0.013*	
C15	0.32741 (10)	0.0188 (2)	0.83746 (18)	0.0111 (6)	
C16	0.38034 (10)	−0.0164 (2)	0.82023 (18)	0.0145 (6)	
H16	0.3878	−0.0954	0.8143	0.017*	
C17	0.42059 (10)	0.0627 (2)	0.81212 (19)	0.0155 (6)	
H17	0.4548	0.0377	0.7998	0.019*	
C18	0.40976 (10)	0.1809 (2)	0.82255 (18)	0.0142 (6)	
H18	0.4371	0.2350	0.8181	0.017*	
C19	0.35893 (10)	0.2184 (2)	0.83938 (19)	0.0136 (6)	
H19	0.3525	0.2978	0.8462	0.016*	
C20	0.31675 (10)	0.1395 (2)	0.84649 (18)	0.0094 (6)	
N1	0.23943 (8)	−0.05699 (18)	0.86829 (15)	0.0098 (5)	
N2	0.14151 (8)	0.00196 (18)	0.90708 (15)	0.0092 (5)	
N3	0.17896 (8)	0.07150 (19)	0.70029 (16)	0.0131 (5)	
N4	0.13255 (9)	0.05323 (19)	0.67637 (16)	0.0136 (5)	
N5	0.08768 (9)	0.0341 (2)	0.65186 (17)	0.0234 (6)	
O1	0.16772 (7)	0.23708 (15)	0.89293 (13)	0.0127 (4)	
O2	0.26797 (6)	0.18073 (15)	0.85790 (12)	0.0113 (4)	
O1W	0.23229 (7)	0.08568 (17)	1.05761 (13)	0.0138 (4)	
O2W	0.0000	−0.0960 (3)	0.7500	0.0383 (8)	
H1W	0.2366 (10)	0.1525 (7)	1.0796 (16)	0.023 (9)*	
H2W	0.2189 (11)	0.0413 (15)	1.0991 (14)	0.037 (10)*	
H3W	0.0224 (11)	−0.056 (3)	0.723 (3)	0.064 (13)*	

### Atomic displacement parameters (Å^2^)

**Table:**

	*U*^11^	*U*^22^	*U*^33^	*U*^12^	*U*^13^	*U*^23^
Mn1	0.0082 (2)	0.0069 (2)	0.0122 (2)	−0.00022 (17)	0.00244 (16)	−0.00015 (16)
C1	0.0133 (14)	0.0136 (14)	0.0037 (13)	0.0011 (11)	0.0004 (10)	0.0013 (11)
C2	0.0163 (14)	0.0085 (14)	0.0124 (14)	0.0000 (11)	0.0008 (11)	0.0017 (11)
C3	0.0190 (15)	0.0117 (15)	0.0162 (15)	0.0046 (12)	0.0017 (12)	0.0029 (11)
C4	0.0105 (14)	0.0172 (16)	0.0271 (16)	0.0050 (12)	0.0059 (12)	0.0031 (13)
C5	0.0130 (14)	0.0117 (15)	0.0239 (16)	−0.0027 (11)	0.0035 (12)	0.0033 (12)
C6	0.0149 (14)	0.0083 (14)	0.0101 (14)	0.0023 (11)	0.0031 (11)	0.0004 (11)
C7	0.0121 (14)	0.0118 (15)	0.0105 (14)	−0.0045 (11)	0.0024 (11)	−0.0004 (11)
C8	0.0129 (14)	0.0080 (14)	0.0064 (13)	−0.0001 (11)	−0.0005 (10)	−0.0019 (10)
C9	0.0119 (14)	0.0114 (15)	0.0138 (14)	−0.0013 (11)	0.0035 (11)	−0.0016 (11)
C10	0.0159 (14)	0.0119 (15)	0.0092 (14)	−0.0046 (11)	0.0014 (11)	0.0001 (11)
C11	0.0205 (15)	0.0067 (14)	0.0093 (13)	0.0021 (11)	−0.0028 (11)	0.0002 (11)
C12	0.0129 (14)	0.0145 (15)	0.0052 (13)	0.0043 (11)	0.0005 (10)	−0.0027 (10)
C13	0.0124 (14)	0.0120 (14)	0.0042 (13)	−0.0028 (11)	−0.0007 (10)	−0.0015 (11)
C14	0.0142 (14)	0.0106 (15)	0.0082 (13)	0.0035 (11)	0.0011 (11)	0.0006 (10)
C15	0.0128 (14)	0.0144 (14)	0.0062 (13)	−0.0007 (11)	0.0016 (10)	0.0010 (11)
C16	0.0170 (15)	0.0162 (15)	0.0104 (14)	0.0038 (12)	0.0021 (11)	0.0012 (11)
C17	0.0069 (14)	0.0278 (17)	0.0119 (14)	0.0028 (12)	0.0011 (11)	0.0015 (12)
C18	0.0107 (14)	0.0240 (17)	0.0077 (14)	−0.0048 (12)	−0.0008 (11)	0.0009 (12)
C19	0.0193 (15)	0.0119 (15)	0.0095 (14)	−0.0023 (12)	−0.0006 (11)	−0.0023 (11)
C20	0.0082 (13)	0.0175 (15)	0.0026 (12)	0.0006 (11)	0.0005 (10)	0.0020 (11)
N1	0.0135 (12)	0.0086 (12)	0.0073 (11)	0.0005 (9)	0.0014 (9)	0.0004 (9)
N2	0.0118 (11)	0.0070 (12)	0.0088 (11)	0.0010 (9)	0.0018 (9)	0.0002 (9)
N3	0.0097 (12)	0.0190 (14)	0.0108 (12)	−0.0020 (9)	0.0013 (9)	0.0014 (9)
N4	0.0198 (14)	0.0138 (13)	0.0078 (12)	0.0027 (10)	0.0047 (10)	0.0002 (9)
N5	0.0122 (13)	0.0395 (17)	0.0183 (13)	0.0007 (12)	−0.0002 (10)	0.0000 (11)
O1	0.0099 (9)	0.0074 (10)	0.0213 (10)	−0.0001 (7)	0.0038 (7)	−0.0005 (8)
O2	0.0114 (9)	0.0097 (10)	0.0133 (10)	−0.0007 (8)	0.0037 (7)	0.0011 (8)
O1W	0.0192 (11)	0.0090 (11)	0.0133 (10)	−0.0039 (8)	0.0027 (8)	−0.0004 (9)
O2W	0.025 (2)	0.028 (2)	0.063 (2)	0.000	0.0130 (18)	0.000

### Geometric parameters (Å, °)

**Table:**

Mn1—O1	1.8636 (18)	C10—H10	0.9300
Mn1—O2	1.8844 (18)	C11—C12	1.379 (3)
Mn1—N2	1.986 (2)	C11—H11	0.9300
Mn1—N1	1.988 (2)	C12—C13	1.393 (3)
Mn1—N3	2.306 (2)	C12—H12	0.9300
Mn1—O1W	2.321 (2)	C13—N1	1.430 (3)
C1—O1	1.324 (3)	C14—N1	1.307 (3)
C1—C2	1.397 (3)	C14—C15	1.427 (3)
C1—C6	1.422 (3)	C14—H14	0.9300
C2—C3	1.377 (3)	C15—C20	1.418 (4)
C2—H2	0.9300	C15—C16	1.420 (3)
C3—C4	1.398 (4)	C16—C17	1.367 (4)
C3—H3	0.9300	C16—H16	0.9300
C4—C5	1.371 (4)	C17—C18	1.392 (4)
C4—H4	0.9300	C17—H17	0.9300
C5—C6	1.411 (3)	C18—C19	1.378 (3)
C5—H5	0.9300	C18—H18	0.9300
C6—C7	1.422 (4)	C19—C20	1.401 (3)
C7—N2	1.301 (3)	C19—H19	0.9300
C7—H7	0.9300	C20—O2	1.330 (3)
C8—C9	1.397 (3)	N3—N4	1.200 (3)
C8—C13	1.401 (3)	N4—N5	1.167 (3)
C8—N2	1.425 (3)	O1W—H1W	0.820 (11)
C9—C10	1.379 (4)	O1W—H2W	0.82 (2)
C9—H9	0.9300	O2W—H3W	0.82 (3)
C10—C11	1.393 (3)		
			
O1—Mn1—O2	90.68 (8)	C11—C10—H10	119.7
O1—Mn1—N2	92.69 (8)	C12—C11—C10	120.0 (2)
O2—Mn1—N2	175.37 (8)	C12—C11—H11	120.0
O1—Mn1—N1	175.33 (8)	C10—C11—H11	120.0
O2—Mn1—N1	93.71 (8)	C11—C12—C13	120.1 (2)
N2—Mn1—N1	82.83 (9)	C11—C12—H12	119.9
O1—Mn1—N3	95.95 (8)	C13—C12—H12	119.9
O2—Mn1—N3	96.55 (7)	C12—C13—C8	119.7 (2)
N2—Mn1—N3	86.26 (8)	C12—C13—N1	125.1 (2)
N1—Mn1—N3	85.12 (8)	C8—C13—N1	115.1 (2)
O1—Mn1—O1W	94.00 (7)	N1—C14—C15	125.5 (2)
O2—Mn1—O1W	88.14 (7)	N1—C14—H14	117.2
N2—Mn1—O1W	88.47 (7)	C15—C14—H14	117.2
N1—Mn1—O1W	84.58 (7)	C20—C15—C16	118.3 (2)
N3—Mn1—O1W	168.94 (7)	C20—C15—C14	125.0 (2)
O1—C1—C2	118.3 (2)	C16—C15—C14	116.6 (2)
O1—C1—C6	123.5 (2)	C17—C16—C15	121.8 (3)
C2—C1—C6	118.2 (2)	C17—C16—H16	119.1
C3—C2—C1	121.3 (2)	C15—C16—H16	119.1
C3—C2—H2	119.4	C16—C17—C18	119.3 (2)
C1—C2—H2	119.4	C16—C17—H17	120.3
C2—C3—C4	120.9 (3)	C18—C17—H17	120.3
C2—C3—H3	119.6	C19—C18—C17	120.6 (2)
C4—C3—H3	119.6	C19—C18—H18	119.7
C5—C4—C3	119.1 (2)	C17—C18—H18	119.7
C5—C4—H4	120.5	C18—C19—C20	121.3 (3)
C3—C4—H4	120.5	C18—C19—H19	119.3
C4—C5—C6	121.4 (2)	C20—C19—H19	119.3
C4—C5—H5	119.3	O2—C20—C19	118.9 (2)
C6—C5—H5	119.3	O2—C20—C15	122.5 (2)
C5—C6—C1	119.2 (2)	C19—C20—C15	118.6 (2)
C5—C6—C7	117.7 (2)	C14—N1—C13	122.8 (2)
C1—C6—C7	123.1 (2)	C14—N1—Mn1	124.04 (18)
N2—C7—C6	125.9 (2)	C13—N1—Mn1	113.16 (16)
N2—C7—H7	117.1	C7—N2—C8	122.2 (2)
C6—C7—H7	117.1	C7—N2—Mn1	124.61 (18)
C9—C8—C13	119.9 (2)	C8—N2—Mn1	112.96 (15)
C9—C8—N2	124.3 (2)	N4—N3—Mn1	117.69 (16)
C13—C8—N2	115.8 (2)	N5—N4—N3	178.8 (3)
C10—C9—C8	119.6 (2)	C1—O1—Mn1	129.44 (16)
C10—C9—H9	120.2	C20—O2—Mn1	128.82 (16)
C8—C9—H9	120.2	Mn1—O1W—H1W	107.2 (17)
C9—C10—C11	120.7 (2)	Mn1—O1W—H2W	123.5 (18)
C9—C10—H10	119.7	H1W—O1W—H2W	114.6 (19)

### Hydrogen-bond geometry (Å, °)

**Table:**

*D*—H···*A*	*D*—H	H···*A*	*D*···*A*	*D*—H···*A*
O1W—H2W···N3^i^	0.82 (2)	2.12 (2)	2.937 (3)	176 (3)
O1W—H1W···O2^ii^	0.82 (1)	2.08 (1)	2.885 (3)	169 (2)
O2W—H3W···N5	0.82 (3)	2.18 (3)	3.000 (3)	173 (4)

Symmetry codes: (i) *x*, −*y*, *z*+1/2; (ii) −*x*+1/2, −*y*+1/2, −*z*+2.
